# Supplementary material for: Modeling of DNA binding to the condensin hinge domain using molecular dynamics simulations guided by atomic force microscopy
Source: PLoS Comput Biol. 2021 Jul 30;17(7):e1009265. doi: 10.1371/journal.pcbi.1009265 (PMC8357123; doi:10.1371/journal.pcbi.1009265)
Supplement: S1 Fig — The Q-score represents how much fraction of the protein/DNA contacts in the crystal structure forms in each simulation snapshot. We considered that protein and DNA particles contacted when these were within 15Å. DNA particle identity was neglected to make this measure robust for DNA sliding. The representative structures with Q-scores of 0.5, 0.8, and 1.0 (the crystal structure) were also presented. (PDF) [file pcbi.1009265.s001.pdf]

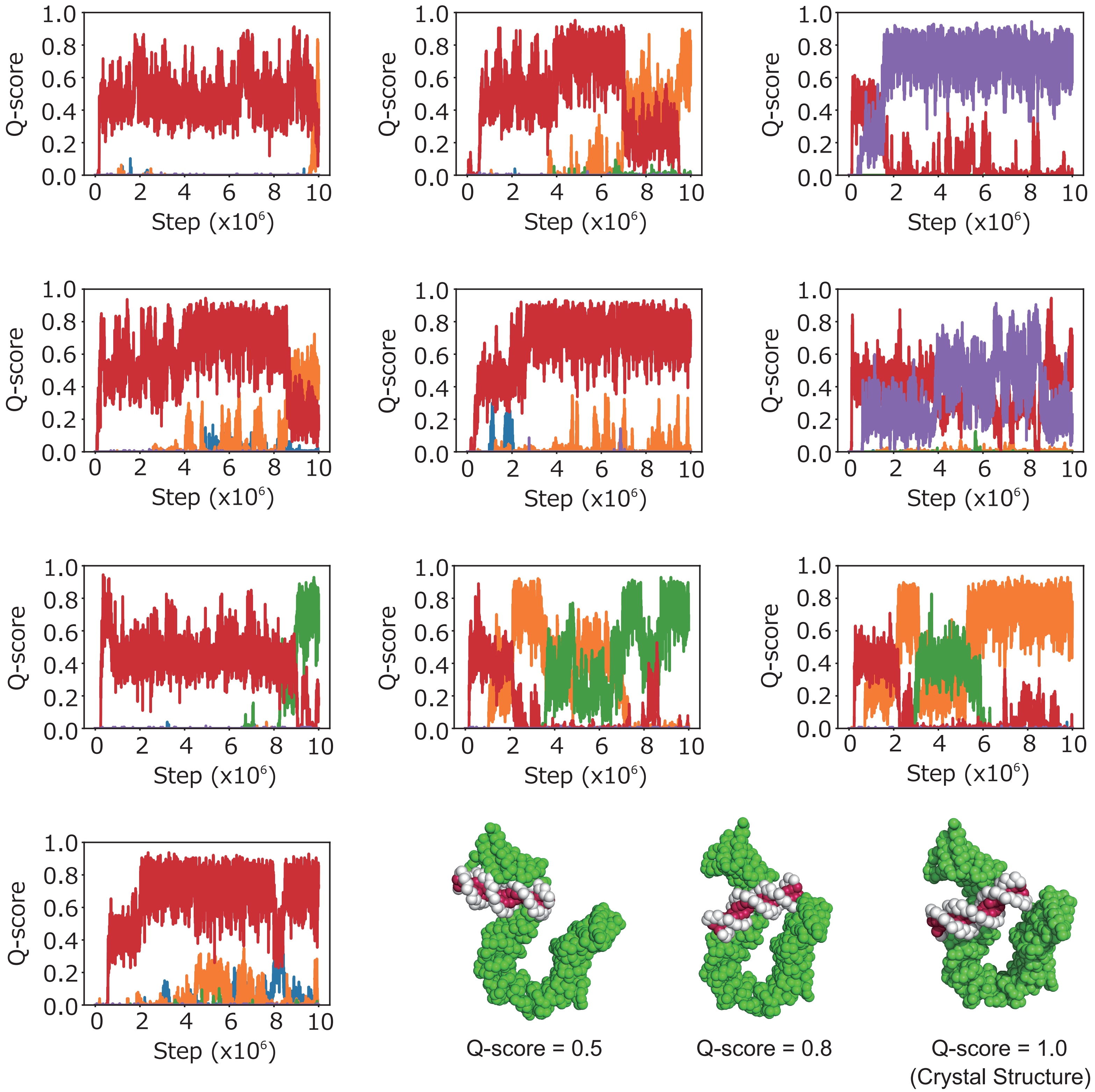

S1 Fig

Time trajectories of Q-score calculated from the Ycg1/DNA complex simulations. The Q-score represents how much fraction of the protein/DNA contacts in the crystal structure forms in each simulation snapshot. We considered that protein and DNA particles contacted when these were within 15Å. DNA particle identity was neglected to make this measure robust for DNA sliding. The representative structures with Q-scores of 0.5, 0.8, and 1.0 (the crystal structure) were also presented.
