# Supplementary material for: Modeling of DNA binding to the condensin hinge domain using molecular dynamics simulations guided by atomic force microscopy
Source: PLoS Comput Biol. 2021 Jul 30;17(7):e1009265. doi: 10.1371/journal.pcbi.1009265 (PMC8357123; doi:10.1371/journal.pcbi.1009265)
Supplement: S2 Fig — (A) The initial structure of CGMD simulations of the Ycs4/dsDNA complex. (B) The representative structures of the CGMD simulation. (C) DNA contact probabilities mapped on the Ycs4 structure. (PDF) [file pcbi.1009265.s002.pdf]

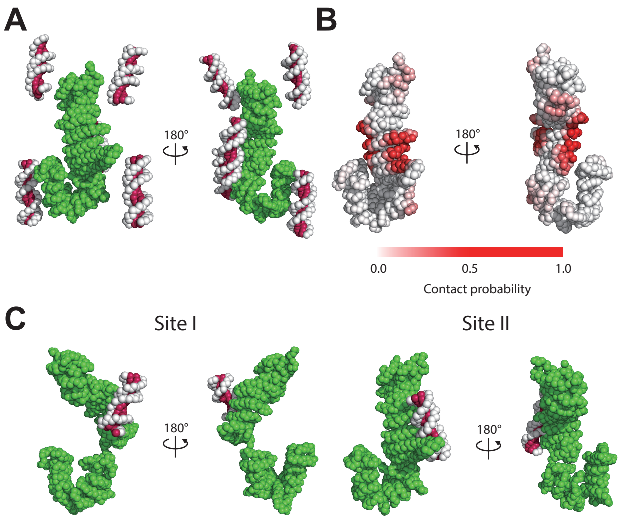

S2 Fig

(A) The initial structure of CGMD simulations of the Ycs4/dsDNA complex. **(B)** The representative structures of the CGMD simulation. **(C)** DNA contact probabilities mapped on the Ycs4 structure.
