# Supplementary material for: Modeling of DNA binding to the condensin hinge domain using molecular dynamics simulations guided by atomic force microscopy
Source: PLoS Comput Biol. 2021 Jul 30;17(7):e1009265. doi: 10.1371/journal.pcbi.1009265 (PMC8357123; doi:10.1371/journal.pcbi.1009265)
Supplement: S3 Fig — In these images, one of the HEAT repeat subunits pointed by the red arrow repeatedly dissociated from and associated to the head domains of Smc2 and Smc4. (PDF) [file pcbi.1009265.s003.pdf]

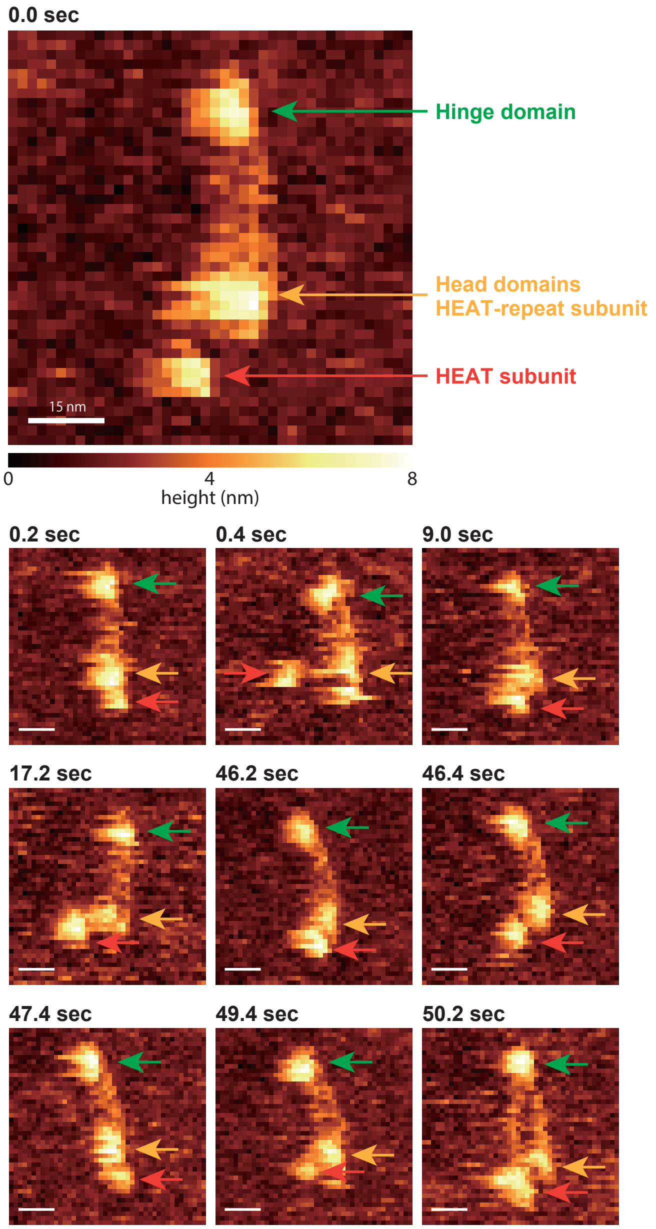

S3 Fig

The representative AFM images of the condensin holo-complex. In these images, one of the HEAT repeat subunits pointed by the red arrow repeatedly dissociated from and associated to the head domains of Smc2 and Smc4.
