# Supplementary material for: Modeling of DNA binding to the condensin hinge domain using molecular dynamics simulations guided by atomic force microscopy
Source: PLoS Comput Biol. 2021 Jul 30;17(7):e1009265. doi: 10.1371/journal.pcbi.1009265 (PMC8357123; doi:10.1371/journal.pcbi.1009265)
Supplement: Attachment — Submitted filename: response_letter.pdf [file pcbi.1009265.s006.pdf]

## Responses to reviewer #1

**Comment 1:** This manuscript combines high-speed atomic force microscopy (hsAFM) and coarse grained molecular dynamics (cgMD) simulations to model double strand DNA (dsDNA) binding to the hinge domain of condensin, which is involved in DNA loop extrusion to compact chromatin during mitosis. The manuscript is well-written and well-organized, but I do have a couple of doubts/concerns detailed below.

We would like to thank reviewer #1 for this encouraging comment. The revisions based on his/her constructive comments below definitely improves the manuscript and would untangle his/her and future readers' concerns.

**Comment 2:** The cgMD simulations sacrifice physical accuracy for length/time-scale expediency, so the initial sanity check of modeling the known closed hinge domain dsDNA-bound crystal structure is well-justified. However, the setup of those simulations and their analysis is not fully satisfying. The removal of Brn1 from the complex in the cgMD simulations changes the comparison, and even if it is necessary to enable dsDNA binding, it could have been included in separate cgMD simulations as a second randomly placed component to see whether the whole complex could be modeled correctly. More importantly, it is not clear to what extent the structure is modeled correctly in the cgMD simulations. The use of minimum distances and principal component analysis and DNA contacts obscures the simple question of how close the model is to the actual structure, which can be answered easily by comparing a coarse-grained RMSD between the model and the crystal structure. This RMSD should be reported as a time series for all 5 dsDNA molecules binding to the condensin hinge domain for all cgMD simulations performed.

We acknowledge that including Brn1 molecules in our simulation system of Ycg1/DNA complex is ideal for fair comparison between our simulations and the Ycg1/Brn1/DNA complex crystal structure. However, it is difficult to achieve for the following two reasons. First, several DNA binding residues in Brn1 is missing in the crystal structure. Our native-structure-based protein model requires this information for reasonable simulations. Second, the topology of the Brn1 binding to DNA makes the conformational sampling almost impossible. In the crystal structure, DNA wrapped around by Brn1 fits into the DNA binding cleft on Ycg1. In simulations, in the worst-case scenario, Brn1 firstly bound to Ycg1 would occupy the DNA binding cleft and inhibit DNA binding to the correct position. To avoid this difficulty, we omitted Brn1 from our simulation

system based on the assumption that Brn1, which binds to DNA with only a few amino acid residues, would play a merely auxiliary role in DNA binding.

In this situation, as reviewer #1 commented, it is important to show what extent the structure is modeled correctly. For this purpose, as Ycg1/DNA interaction is non-DNA-sequence-specific, we need a structure similarity measure that is robust for DNA sliding, i.e. the value keeps low even if DNA slides by a few base-pairs along the DNA binding cavity on Ycg1. Thus, instead of RMSD, which is not robust for DNA sliding, we calculated Q-score for each simulation snapshot and plotted it as a function of time in the new Figure S1. The Q-score represents how much fraction of the protein/DNA contacts in the crystal structure forms in each simulation snapshot. We considered that protein and DNA particles contacted when these were within 10Å. DNA particle identity was neglected to make this measure robust for DNA sliding. The plots revealed that, as the simulations proceed, Q-score increased as high as 0.9 and fluctuated between 0.5 and 0.9, i.e. 50-90% of the protein/DNA contacts in the crystal structure forms in the simulation snapshots. This result partially justifies the omission of Brn1 since the structure can be predicted in a certain degree of accuracy without it. The addition of this new figure makes significant improvement of the manuscript and readers should get insight into what extent the structure is modeled correctly. We would like to thank reviewer 1 for this constructive suggestion.

---

**Revision for comment 2:** We added the sentence “This topology makes conformational sampling of DNA binding to the Ycg1/Brn1 complex by CGMD simulations almost impossible. As Brn1, which binds to DNA with a few amino acid residues, would play a merely auxiliary role in DNA binding, we neglected Brn1 and conducted the blind prediction of the Ycg1/dsDNA complex structure.” to line 119 in page 6. We also added Fig. S1 and a paragraph “To get insight into how similar the structures in the simulations are to the crystal structure, we calculated Q-score for each simulation snapshot and plotted it as a function of time (Fig. S1). The Q-score represents how much fraction of the protein/DNA contacts in the crystal structure forms in each simulation snapshot. We considered that protein and DNA particles contacted when these were within 10Å. DNA particle identity was neglected to make this measure robust for DNA sliding. The plots reveal that, as the simulations proceed, Q-score increased as high as 0.9 and fluctuated between 0.5 and 0.9, i.e. 50-90% of the protein/DNA contacts in the crystal structure forms in the simulations.” to line 265 in page 13.

---

**Comment 3:** The other concern I have is the difficulty for a reader of this manuscript to judge what other structures are presently available for condensin hinge domains and how they relate to the cgMD simulations in this manuscript. I think the best way to address this is to show all the known structures in a side-by-side visual depiction in the coarse-grained format, and also to provide coarse-grained RMSD time series analysis for proximity to each known structure for the cgMD simulations.

Unfortunately, we do not have enough structural information to accomplish this fascinating analysis. For budding yeast condensin, which we explore in this study, the crystal structure of the hinge in the closed conformation was reported. The recently reported cryo-EM structure also contains the hinge in the closed conformation. The open hinge conformation was observed only in atomic force microscopy (AFM) imaging. Indeed, this is the reason why we try to model the open hinge conformation based on the AFM image in the current study. We acknowledge that this discussion should be clearly written to improve the manuscript.

**Revision for comment 3:** We added the sentence "for budding yeast condensin, the structures of the closed hinge in the closed conformation were reported (18, 25), while the open hinge conformation has been observed only in AFM imaging." to line 86 in page 5.

**Comment 4:** Finally, the coarse-grained 3D coordinates for a predicted model for the DNA-bound open hinge domain should be provided in the supplementary material.

We fully agree with this comment.

**Revision for comment 4:** We added the supplementary material.

## Responses to reviewer #2

**Comment 1:** The manuscript presents a computational study on some structural aspects of the SMC and predicts the binding between condensin and DNA. A coarse-grained model is used which is calibrated against AFM data. The study contributes to this interesting topic and is very timely.

It is clearly written and the figures are elegant. There are some methodological aspects that are not clear that require some clarifications to make the manuscript easy to read and to apply its approach to other systems.

We would like to thank reviewer #2 for this overall positive comment. The following comments are also helpful to clarify and significantly improve the manuscript.

**Comment 2:** In the modeling of the interactions between Ycg1 and DNA (Figure 1), the protein seems to be of the same conformations. Is it modeled as a flexible molecule? How much the flexibility affects its binding to DNA?

Yes, the protein (AICG2+) model used in this study allow the fluctuation (RMSD ~ 3Å) around its native reference structure. The 2.2Å RMSD between DNA bound and unbound crystal structures suggests that the effect of protein flexibility to DNA binding is minor, if any.

**Revision for comment 2:** We added the sentence "the 2.2Å root mean square displacement between DNA bound and unbound crystal structures suggests that the effect of protein flexibility to DNA binding is minor, if any." to line 122 in page 6.

**Comment 3:** In the modeling of the open hinge, how many AFM images were included? Was the fitting of the computational model done to a single AFM image?

Yes, the fitting was done to a single AFM image. We chose the image capturing the hinge with 79° hinge angle based on the assumption that DNA would tend to bind to the most open hinge.

**Revision for comment 3:** We added the sentence "For the fitting, we chose a single AFM image of the hinge with 79° hinge angle based on the assumption that DNA would tend to bind to the most open hinge." to line 341 in page 16.

**Comment 4:** In the modeling of the open hinge structure, the modeling using kappa is unclear and more details may help. Was this transition modeled just by a single parameter? Also, choosing

the value of kappa is unclear. The differences between the results for k=10, 3000, 10000 are very small (Figure 3C). The difference between them are of just less than 10 simulations while the other >200 simulations behave very similarly? Are the simulations sensitive to kappa? Also in figure 3C, k should be changed to kappa.

In this modeling, the molecules, Smc2 and Smc4, are under the effect of the AFM fitting potential and the native-structure-based potential. These two potentials are to stabilize the open conformation observed in the AFM image and the closed conformation in the crystal structure, respectively. The $\kappa$ is the only controllable parameter that decides the stability balance between the open and closed conformation. In the most simulations, regardless of the $\kappa$ value, the molecules diffused away and got out of reach of the AFM potential. However, in a few cases with $\kappa$ set to 3000, the molecules are fitted into the AFM potential. Importantly, once fitted, the molecules never left from the positions. When $\kappa$ was set to 10000, the potential tended to hold the molecules in the AFM image region, but, in some cases, distorted each molecular structure, suggesting too strong force from the AFM fitting potential. Thus, the simulations were sensitive to the value of $\kappa$ to some extent. However, the small deviation of $\kappa$ (for example from 1000 to 5000) did not cause the significant change of the results as stated in the manuscript.

**Revision for comment 4:** We added the sentence “In the subsequent flexible-fitting, the molecules, Smc2 and Smc4, are under the effect of the AFM fitting potential and the native-structure-based potential. These two potentials are to stabilize the open conformation observed in the AFM image and the closed conformation in the crystal structure, respectively. The $\kappa$ is the only controllable parameter that decides the stability balance between the open and closed conformation.” to line 350 in page 17. We also added the sentence “Regardless of the κ value, in most of the simulations, the Smc2 and Smc4 molecules diffused away from each other and got out of reach of the AFM potential (Fig. 3C top; nearly zero inter-subunit Q-score).” to line 363 in page 17.

**Comment 5:** In general, the adding more details to the figure legends may help to understand the figures.

We fully agree with this comment.

**Revision for comment 5:** We added one or more sentences to each of all the figure legends.

**Comment 6:** In some simulations, the interactions between the DNA was studied with open or closed hinge. However, Figure 5 presents a transition from the open to closed. How was this modeled? Also with kappa? If so, how sensitive this to the value of kappa?

In this simulation, the initial structure was the open hinge conformation modeled using the AFM potential with kappa set to 3000. Then, we performed simulations without the AFM fitting potential. Then, the structure relaxes under the effect of the native-structure-based potential that stabilizes the reference closed hinge conformation.

**Revision for comment 6:** We added the sentence “Then, we performed simulations without the AFM fitting potential. In this simulation, the structure relaxes under the effect of the native-structure-based potential that stabilizes the reference closed hinge conformation.” to line 440 in page 21.
